# Ray‐Tracing‐Based Intraocular Lens Power Calculation in Combined Cataract Surgery and Descemet Membrane Endothelial Keratoplasty

**DOI:** 10.1111/ceo.70135

**Published:** 2026-06-23

**Authors:** Jan O. Weber, Klemens P. Kaiser, Jascha A. Wendelstein, H. Burkhard Dick, Thomas Kohnen, Ingo Schmack

**Affiliations:** ^1^ Department of Ophthalmology Goethe‐University Frankfurt Germany; ^2^ Department of Ophthalmology University Hospital Munich, Ludwig‐Maximilian‐University Munich Germany; ^3^ Institute for Refractive and Ophthalmic Surgery (IROC) Zurich Switzerland; ^4^ Department of Experimental Ophthalmology Saarland University Homburg Germany; ^5^ Knappschaftskrankenhaus Eye Clinic, University Hospital of the Ruhr University Bochum Bochum Germany

**Keywords:** Barrett universal II, cataract surgery, Descemet membrane endothelial keratoplasty, Fuchs endothelial corneal dystrophy, intraocular lens power calculation, ray tracing, triple DMEK

## Abstract

**Background:**

To compare the predictive accuracy of ray tracing‐based intraocular lens (IOL) power calculation formulas with the Barrett Universal II (BUII) formula in eyes with Fuchs endothelial corneal dystrophy (FECD) undergoing combined cataract surgery and Descemet membrane endothelial keratoplasty (triple DMEK).

**Methods:**

This is a retrospective, single‐centre comparative case series. Preoperative biometry was obtained using Scheimpflug tomography (Pentacam AXL) and a swept‐source optical coherence tomography–based biometer (IOLMaster 700). IOL power calculations were performed using Barrett Universal II (BUII), Olsen ray‐tracing and two OKULIX ray‐tracing approaches (paraxial and 2.5‐mm pupil). Prediction error (PE) was calculated and trueness, precision, and accuracy were evaluated using robust statistical methods (Eyetemis). A retrospective IOLup1D adjustment was additionally assessed.

**Results:**

One hundred forty‐nine eyes of 149 patients were enrolled. BUII demonstrated the lowest trimmed mean absolute error (0.74 ± 0.65D) and median absolute error (0.60D), with 72% of eyes within ±1.00D of the target refraction. Olsen ray‐tracing showed higher errors (mean absolute error 1.06 ± 1.02D), while both OKULIX approaches performed similarly (0.91–0.92D). Only OKULIX paraxial ray‐tracing showed no significant systematic bias (*p* = 0.450), whereas all other formulas demonstrated a significant hyperopic shift (*p* < 0.001). After retrospective IOLup1D adjustment, BUII and Olsen achieved comparable accuracy, with Olsen demonstrating the highest trueness. Mean keratometry values measured with the IOLMaster were 0.20 ± 0.63D steeper compared to the Pentacam.

**Conclusion:**

In triple DMEK eyes, ray tracing–based IOL power calculation did not offer better accuracy compared to BUII. BUII demonstrated the highest accuracy and precision overall. After IOLup1D adjustment, BUII and Olsen achieved comparable accuracy, with Olsen showing the greatest trueness.

## 1 Introduction

Fuchs endothelial corneal dystrophy (FECD) is a leading cause of corneal endothelial decompensation and is commonly treated with Descemet membrane endothelial keratoplasty (DMEK), in which the diseased endothelium and Descemet membrane are selectively replaced by donor tissue [1]. Owing to rapid visual rehabilitation, favourable refractive stability and low graft rejection rates, DMEK has become the preferred surgical technique for FECD [1, 2, 3].

A substantial proportion of FECD patients present with clinically significant cataract at the time of surgery. In such cases, combined phacoemulsification with intraocular lens (IOL) implantation and DMEK (“triple DMEK”) is frequently performed, allowing simultaneous management of corneal endothelial pathology and opacities of the crystalline lens [2, 4, 5]. However, several studies have reported a postoperative hyperopic shift following triple DMEK surgery, resulting in deviation from the intended refractive target [6, 7, 8].

Accurate IOL power calculation is therefore critical in this patient population [4, 5, 6, 7, 8]. While modern‐generation formulas such as the Barrett Universal II (BUII) have demonstrated good overall performance, they rely on simplified corneal power estimations based primarily on anterior corneal curvature [9, 10]. Ray‐tracing–based formulas incorporate measured anterior and posterior corneal curvature and corneal thickness, potentially offering improved accuracy in eyes with altered corneal anatomy, such as keratoconus or FECD. After a triple Descemet membrane endothelial keratoplasty (triple DMEK) surgery, the corneal anatomy typically undergoes structural changes characterised by a reduction in corneal oedema, thinning of the central cornea, and restoration of posterior corneal curvature. These changes can affect both anterior and posterior corneal surfaces and may alter shifts in corneal power and refractive outcomes during the postoperative healing phase [6, 11]. The OKULIX Ray‐tracing software builds a specific optical model of patients' eyes using biometric and corneal topography or tomography data and simulates the distribution of light rays through the ocular media to determine retinal image quality and the optimal IOL power [12, 13]. In contrast to conventional formula‐based methods, the algorithm incorporates a full optical model of the eye, including measurements of the anterior and posterior corneal surfaces, central corneal thickness (CCT), axial length, anterior chamber depth (ACD) and white‐to‐white distance, thereby enabling a more individualised and physics‐based IOL power prediction [10]. However, it remains unclear whether ray‐tracing‐based formulas provide clinically meaningful advantages over modern vergence‐based formulas in triple DMEK eyes.

This study seeks to evaluate the performance of the ray‐tracing formulas for IOL power calculation in FECD eyes undergoing triple DMEK. Their predictive accuracy was compared with the BUII formula to determine whether ray‐tracing–based IOL calculation provides an advantage in this challenging clinical setting.

## 2 Methods

In this retrospective, single‐centre, consecutive case series, the refractive outcomes of patients who underwent triple‐DMEK for FECD between January 2017 and July 2023 at the Department of Ophthalmology, Goethe‐University Frankfurt, Germany, were analysed. Ethical approval for this study was obtained from the Ethics Committee of the Faculty of Medicine at Goethe University Frankfurt, Germany (approval number 2026‐2842). All procedures were conducted in accordance with the tenets of the Declaration of Helsinki.

### 2.1 Inclusion and Exclusion Criteria

This study enrolled eyes of patients aged ≥ 18 years who underwent triple DMEK for FECD‐related corneal endothelial dysfunction. Eyes with previous ocular surgery, relevant concomitant ocular pathology affecting visual acuity or IOL power calculation, or incomplete preoperative biometry or refraction data were excluded. Furthermore, eyes with a follow‐up of < 3 months and a postoperative CDVA of > 0.3 logMAR were excluded as recommended by guidelines [14].

### 2.2 Preoperative and Postoperative Assessment

All eyes underwent a comprehensive preoperative medical evaluation, as well as preoperative biometry by Scheimpflug‐technology and partial optical coherence interferometry using Pentacam AXL or AXL Wave (OCULUS Optikgeräte GmbH, Wetzlar, Germany) including tomography values, CCT, ACD, lens thickness (LT) and AL. In cases where axial length (AL) measurements were incomplete or absent, AL was obtained by the IOLMaster 700 (Carl Zeiss Meditec AG, Jena, Germany). In addition, preoperative and postoperative subjective refraction was obtained by qualified optometrists. Assessment of postoperative subjective refraction was performed at least 3 months after surgery to ensure the stabilisation of the refraction.

### 2.3 IOL Power Calculation

All IOL power calculations and corresponding predicted refractions were performed using Pentacam software. OKULIX (Tedics Peric & Jöher GbR, OKULIX software version V9.35.) calculations were performed with measurements from the Pentacam. The BUII and Olsen ray‐tracing formulas were calculated directly using the latest available Pentacam software version (1.30r04). An A‐constant of 118.8 for the SA60AT/SN60AT IOL (Alcon) was obtained from the Pentacam IOL Calculator database and applied in the BUII formula. For Olsen, the ACD constant was set to 4.57. The OKULIX ray‐tracing method utilises IOL‐specific geometry data. The prediction error (PE) was calculated as the difference between the actual postoperative spherical equivalent (SEQ) at the spectacle plane (vertex distance = 12 mm) and the predicted refraction based on the implanted IOL power. In the PE interpretation, a positive value indicates a hyperopic shift, and a negative value indicates a myopic shift.

Furthermore, an additional calculation was performed using the IOLup1D method, as described by Khan et al. This approach accounts for the systematic hyperopic shift that occurs after DMEK by simulating the selection of an IOL power 1.0 D stronger than formula‐recommended. Since this was a retrospective study, the method was applied in reverse: the implanted IOL power was reduced by 1.0 D, and the corresponding target refraction was used to calculate the PE [5, 15, 16].

### 2.4 IOL and Surgical Technique

Only eyes with implantation of a single‐piece, monofocal, hydrophobic IOL model (AcrySof SA60AT or SN60AT; Alcon) were included, as proposed by Hoffer and associates, to avoid using different IOL constants in the calculation process [17]. The monofocal AcrySof SA60AT IOL is a hydrophobic acrylic IOL with an optical diameter of 6.0 mm and an overall diameter of 13.0 mm. In comparison, the AcrySof SN60AT IOL has the same characteristics and also features a blue light filter. Generally, surgeons aimed for an additional myopia of approximately −0.75 dioptres (D) to −1.0 D when selecting the IOL power for triple DMEK. Combined cataract and DMEK procedures involved phacoemulsification via a clear corneal incision, followed by IOL implantation into the capsular bag, then removal and replacement of the diseased recipient Descemet membrane (DM) and endothelium with a healthy donor lamella comprising DM and corneal endothelium. Surgeries were performed by two experienced surgeons (TK, IS) at the Department of Ophthalmology, Goethe University, Frankfurt, Germany, using peribulbar or general anaesthesia.

### 2.5 Outcome Measures

Primary outcome measures were mean PE (MPE), trimmed mean absolute error (tMAE), median absolute error (MedAE), root mean squared PE (RMSE), standard deviation (SD), and the minimum and maximum of those errors, as well as the percentages of eyes within ±0.50 D, ±1.00 D, ±1.50 D, and ±2.00 D of the predicted target refraction. Outcome measures were determined and evaluated for each formula.

### 2.6 Statistical Analysis

Patients with two eyes meeting the inclusion criteria had only one eye randomly selected for statistical analysis. The data were entered manually into an Excel spreadsheet (version 16.86; Microsoft Corporation). SEQ‐PE values were analysed using the Eyetemis online tool by Kan‐Tor et al. (www.Eyetemis.com), using the “paired SEQ‐PE” option [18].

Trueness is defined as the extent to which measurement errors approach zero, assessed using a one‐sample robust *t*‐test for within‐group comparisons and a two‐sample robust *t*‐test for between‐group comparisons. Precision measures the variability of repeated observations and is evaluated by comparing pairwise differences of SEQ‐PEs between groups using the two‐sample robust *t*‐test. Accuracy is determined using a two‐sample robust *t*‐test comparing absolute SEQ‐PEs across groups, with further analysis using Cochran's Q test and the McNemar test to assess significant differences within predefined thresholds [18, 19].

Eyetemis analysis set‐up: The following settings were used to analyse the data set with the latest online version available assessed in December 2025. *p*‐value adjustment method: Holm; alpha value: 0.05; PE thresholds: ±0.50, ±1.00 D, ±1.50 D, ±2.00 D; study lane length (m): 6.0; adjusted lane length (m): 6.0.

## 3 Results

The study included 149 eyes of 149 patients that underwent triple DMEK procedure. Demographics and biometric characteristics are summarised in Table 1.

**TABLE 1:** Demographic and biometric data of the study population.

Parameter	Mean ± SD	Range
Patients (*n* = 149)		
Age (years)	66.58 ± 9.22	33.0 to 85.0
Sex (female)	93 (62.4%)	
Right eyes	115 (77.2%)	
Eyes (*n* = 149)		
Mean keratometry (D)	43.33 ± 1.61	38.60 to 48.0
Mean posterior keratometry (D)	5.85 ± 0.49	3.90 to 6.80
Q_a_‐value	−0.33 ± 0.22	−0.99 to 0.57
Q_p_‐value	0.06 ± 0.61	−4.36 to 2.32
CCT (μm)	619.22 ± 83.23	476 to 806
Internal ACD (mm)	2.53 ± 0.40	1.35 to 3.96
AL (mm)	23.86 ± 1.28	21.31 to 28.06
IOL power implanted (D)	21.35 ± 3.51	13 to 25
Postop. SEQ (D)	−0.46 ± 1.10	−4.75 to 2.5
Postop. CDVA (logMAR)	0.21 ± 0.26	−0.1 to 1

Abbreviations: ACD, anterior chamber depth; AL, axial length measured with IOL Master 700; CCT, central corneal thickness; CDVA, corrected distance visual acuity; D, diopters; IOL, intraocular lens; Q_a_‐value, asphericity coefficient of the anterior corneal surface; Q_p_‐value, asphericity coefficient of the posterior corneal surface; SD, standard deviation; SEQ, spherical equivalent.

Median PE, MPE, tMAE, MedAE and RMSE for conventional calculations and for the IOLup1D method are shown in Table 2.

**TABLE 2:** Refractive absolute and arithmetic prediction errors (median, mean ± SD) and root mean square (RMSE).

	MedAE (D)	tMAE ± SD (D)	MedPE ± SD (D)	MPE ± SD (D)	RMSE (D)
BUII	0.60	0.74 ± 0.65	0.23	0.35 ± 0.92	0.98
Olsen Ray‐tracing	0.81	1.06 ± 1.02	0.66	0.87 ± 1.18	1.47
OKULIX RT Parax	0.68	0.91 ± 0.85	−0.02	0.17 ± 1.24	1.25
OKULIX RT Pup 2.5	0.63	0.92 ± 0.89	0.26	0.39 ± 1.22	1.28
BUII IOLup1D	0.65	0.81 ± 0.59	−0.51	−0.39 ± 1.00	1.00
Olsen Ray‐tracing IOLup1D	0.67	0.86 ± 0.82	−0.09	0.14 ± 1.18	1.19
OKULIX RT Parax IOLup1D	0.92	1.10 ± 0.77	−0.72	−0.51 ± 1.24	1.34
OKULIX RT Pup 2.5 IOLup1D	0.83	0.99 ± 0.77	−0.46	−0.30 ± 1.22	1.26

*Note:* OKULIX Parax = this calculation is based on paraxial optics (Gaussian optics). OKULIX Pup 2.5 = this refers to a calculation in which a pupil diameter of 2.5 mm is simulated.

Abbreviations: BUII, Barrett Universal II advanced IOL calculation formula; D, diopters; IOL, intraocular lens; MedAE, median absolute error; MedPE, median arithmetic prediction error; MPE, mean arithmetic prediction error; Olsen, Olsen Ray‐tracing formula; RT, ray‐tracing; SD, standard deviation; tMAE, trimmed mean absolute error.

Subgroup analysis stratified by preoperative CCT revealed a trend towards increasing hyperopic prediction error with greater corneal oedema across all formulas; in eyes with CCT ≥ 700 μm (*n* = 9), mean prediction errors were highest for Olsen ray‐tracing (+1.95 ± 1.47 D) and BUII (+0.91 ± 0.99 D), whereas both OKULIX approaches showed lower systematic bias in this subgroup (Table 3).

**TABLE 3:** Subgroup analysis of prediction errors stratified by preoperative central corneal thickness (CCT). Arithmetic and absolute prediction errors are reported per formula across three CCT subgroups.

Formula	MPE ± SD (D)	MedAE (D)	tMAE ± SD (D)	±0.50 D (%)	±1.00 D (%)	*p* ^a^
CCT < 600 μm (*n* = 59)						
BUII	+0.16 ± 0.76	0.42	0.54 ± 0.55	54.2	88.1	0.111
Olsen Ray‐tracing	+0.52 ± 0.86	0.57	0.73 ± 0.69	47.5	78.0	< 0.001
OKULIX RT Pup 2.5	−0.33 ± 1.20	0.66	0.96 ± 0.78	39.0	61.0	0.041
OKULIX RT Parax	−0.15 ± 1.03	0.71	0.79 ± 0.67	40.7	67.8	0.255
CCT 600–699 μm (*n* = 80)						
BUII	+0.44 ± 0.99	0.70	0.84 ± 0.68	41.3	61.3	< 0.001
Olsen Ray‐tracing	+1.03 ± 1.26	1.06	1.22 ± 1.08	28.8	47.5	< 0.001
OKULIX RT Pup 2.5	+0.20 ± 1.27	0.59	0.96 ± 0.85	36.3	62.5	0.157
OKULIX RT Parax	+0.35 ± 1.33	0.64	0.98 ± 0.96	36.3	63.8	0.020
CCT ≥ 700 μm (*n* = 9)						
BUII	+0.91 ± 0.99	0.98	1.13 ± 0.68	22.2	66.7	0.025
Olsen Ray‐tracing	+1.95 ± 1.47	1.63	1.95 ± 1.47	0.0	22.2	0.004
OKULIX RT Pup 2.5	+0.57 ± 0.90	0.83	0.89 ± 0.53	33.3	66.7	0.092
OKULIX RT Parax	+0.84 ± 1.31	1.23	1.20 ± 0.94	33.3	44.4	0.093

Abbreviations: BUII, Barrett Universal II; CCT, central corneal thickness; D, diopters; MedAE, median absolute error; MPE, mean prediction error; RT, ray‐tracing; SD, standard deviation; tMAE, trimmed mean absolute error.

^a^ One‐sample *t*‐test; *p* < 0.05 indicates significant systematic bias.

No statistically significant difference was found in CCT (mean difference 1.79 μm; *p* = 0.559) between the IOLMaster 700 and Pentacam. However, mean keratometry differed significantly with steeper values measured using the IOLMaster 700 (mean difference 0.20 D; *p* < 0.001), as depicted in Table 4. Table 5 shows the pre‐ to postoperative differences in mean keratometry values derived from the anterior and posterior corneal surfaces, as well as in total corneal power.

**TABLE 4:** Differences in arithmetic (Δ) and absolute (|Δ|) mean keratometry (K_m_) between measurements obtained with the IOLMaster 700 and Pentacam, including proportions within predefined difference ranges.

	ΔK_m_	|Δ| K_m_
Mean	0.20	0.47
SD	0.63	0.47
Min	−2.32	0.00
Max	2.82	2.82
≥ 2 D	0.7%	2.7%
≥ 1 D and < 2 D	5.4%	7.4%
≥ 0.5 D and < 1 D	17.4%	21.5%
≥ 0 D and < 0.5 D	48.3%	68.5%
< 0 D and ≥ −0.5 D	20.1%	—
< −0.5 D and ≥ −1 D	4.0%	—
< −1 D and ≥ −2 D	2.0%	—
< −2 D	2.0%	—

Abbreviations: D, diopters; Max, maximum; Min, minimum; SD, standard deviation.

**TABLE 5:** Pre‐ and post‐operative differences (Δ) in Pentacam‐derived mean keratometry (K_m_) of the anterior and posterior corneal surfaces and total corneal power (TCP), including the distribution of eyes within predefined difference ranges.

	ΔK_m_ anterior (D)	Δ K_m_ posterior (D)	Δ TCP (D)
Mean	0.22	0.43	0.80
SD	0.59	0.73	1.13
Min	−1.20	−2.85	−1.96
Max	3.00	4.16	6.44
≥ 2 D	2.0%	2.0%	10.7%
≥ 1 D and < 2 D	5.4%	9.4%	14.8%
≥ 0.5 D and < 1 D	11.4%	27.5%	28.9%
≥ 0 D and < 0.5 D	40.3%	37.6%	25.5%
< 0 D and ≥ −0.5 D	30.9%	16.8%	12.1%
< −0.5 D and ≥ −1 D	6.0%	0.7%	2.7%
< −1 D and ≥ −2 D	2.0%	1.3%	1.3%
< −2 D	0.0%	0.7%	0.0%

Abbreviations: D, diopters; Max, maximum; Min, minimum; SD, standard deviation.

**FIGURE 1:**
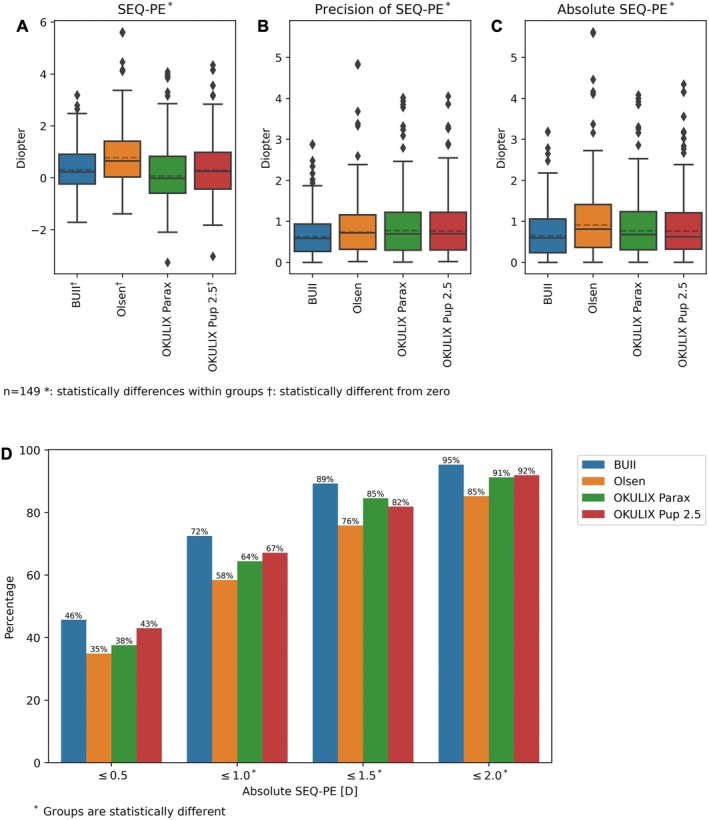
Comprehensive statistical analysis of the tested formulas for all eyes (*n* = 149). (A) The trueness of spherical equivalent prediction errors (SEQ‐PE); (B) the precision; (C) the accuracy; and (D) the percentage of eyes within a specific diopter range of all formulas. BUII, Barrett Universal II; D, diopters.

Figure 1 illustrates trueness (Figure 1A), precision (Figure 1B), accuracy (Figure 1C), and the proportions of eyes within predefined absolute PE thresholds (Figure 1D).

Accuracy differed significantly among formulas. BUII demonstrated the lowest absolute prediction error and was significantly more accurate than Olsen (adjusted *p* = 0.002), OKULIX paraxial (adjusted *p* = 0.006), and OKULIX 2.5‐mm pupil (adjusted *p* = 0.002). Olsen exhibited significantly higher absolute prediction errors compared with OKULIX paraxial (adjusted *p* = 0.021) and OKULIX 2.5‐mm pupil (adjusted *p* = 0.005), indicating lower accuracy.

No statistically significant difference was observed between the two OKULIX approaches (adjusted *p* = 0.818).

Pairwise comparisons of absolute PE also demonstrated significant differences in accuracy between most formulas (*p* < 0.001), whereas no significant difference was observed between the two OKULIX approaches (*p* = 0.818). Overall, BUII demonstrated the highest accuracy compared with the other formulas.

Regarding trueness, OKULIX using a paraxial model was the only formula without a significant systematic bias, with PEs not significantly different from zero (*p* = 0.450). In contrast, BUII, OKULIX 2.5‐mm pupil, and Olsen exhibited significant hyperopic PEs (all *p* < 0.001), with Olsen demonstrating the lowest trueness. Precision differed among formulas: BUII showed the highest precision with the narrowest spread of signed prediction errors (SEQ‐PEs), followed by both OKULIX approaches, which showed comparable moderate variability. Again, Olsen exhibited the largest spread, indicating the lowest precision.

**FIGURE 2:**
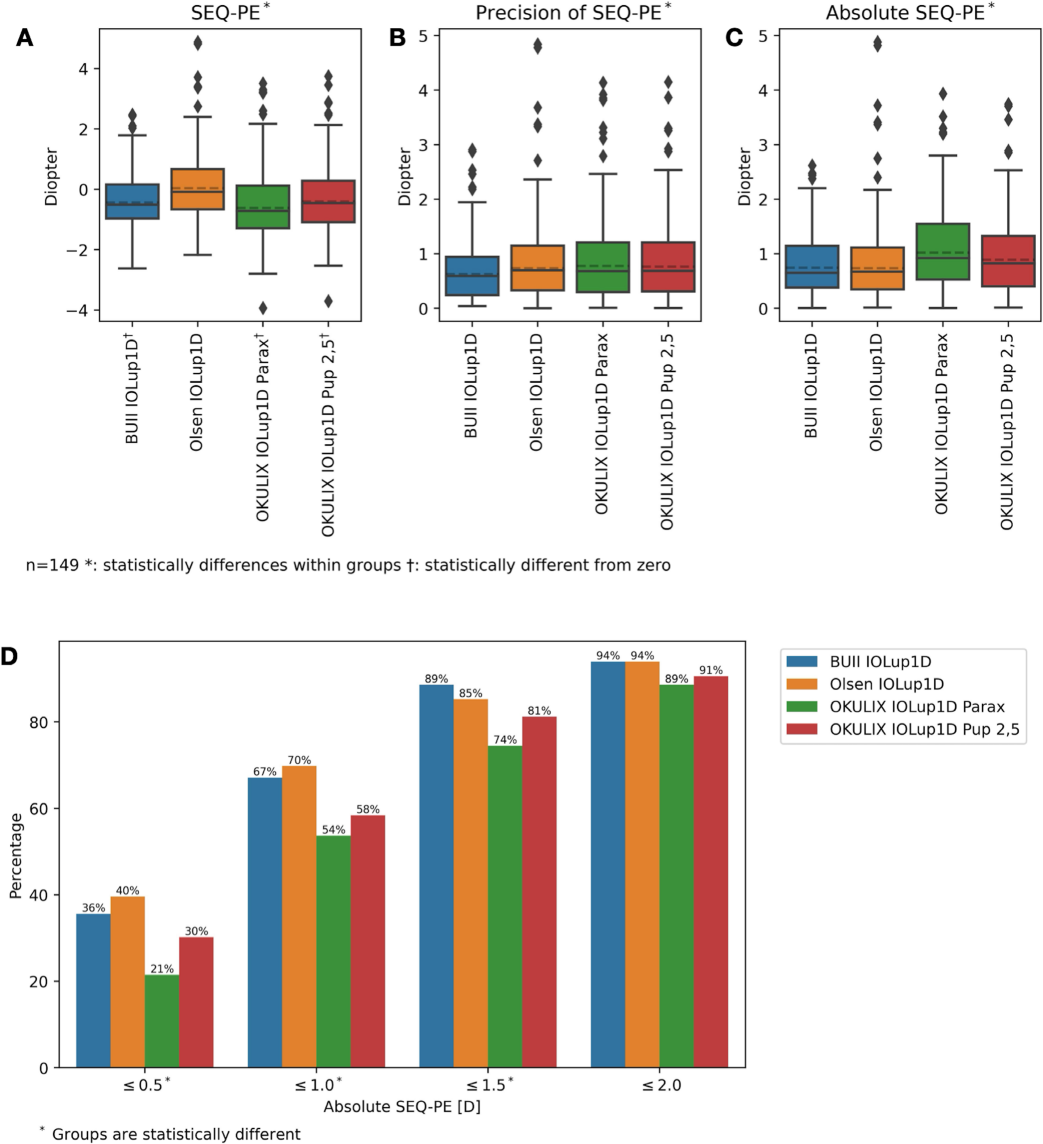
Comprehensive statistical analysis of the tested formulas using the IOLup1D method for all eyes (*n* = 149). (A) The trueness of spherical equivalent prediction errors (SEQ‐PE); (B) the precision; (C) the accuracy; and (D) the percentage of eyes within a specific diopter range of all formulas. BUII, Barrett Universal II; D, diopters.

BUII showed the highest proportion of eyes within target refraction across all absolute prediction error thresholds compared with the ray‐tracing formulas, as illustrated in Figures 1D and 2. Supporting Information shows graphs of the PE and the pre‐ and postoperative differences in total corneal power, anterior simulated keratometry, and posterior simulated keratometry for all four formulae.

### 3.1 IOLup1D

For IOLup1D analysis, accuracy patterns differed slightly (Figure 2D). At the ±0.50 D threshold, Olsen achieved the highest accuracy, followed by BUII, OKULIX 2.5‐mm pupil and OKULIX paraxial. Within ±1.00 D, Olsen and BUII showed comparable performance and outperformed both OKULIX approaches. At broader thresholds, BUII consistently demonstrated the highest accuracy, as illustrated in (Figure 2D).

In IOLup1D analysis (Table 2), Olsen and BUII achieved the highest accuracy. Within ±1.00 D, 69.8% of eyes were within target refraction using Olsen and 67.1% using BUII. BUII demonstrated the lowest median (0.65 D) and mean absolute error (0.81 ± 0.59 D), whereas Olsen showed the least systematic bias (median arithmetic error −0.09 D). OKULIX 2.5‐mm pupil outperformed OKULIX paraxial but remained inferior to BUII and Olsen.

Pairwise comparisons of signed prediction errors are shown in Table 6. For the IOLup1D method, OKULIX paraxial differed significantly from all other formulas (*p* ≤ 0.03), while BUII and Olsen showed no significant difference (*p* = 0.892). OKULIX 2.5‐mm pupil differed significantly from both BUII and Olsen (*p* ≤ 0.03).

**TABLE 6:** *p*‐values for comparisons between formulas.

Formula	*p*‐values			
	BUII	Olsen ray‐tracing	OKULIX RT Parax	OKULIX RT Pup 2.5
BUII	—	< 0.001	< 0.001	0.856
Olsen ray‐tracing	< 0.001	—	< 0.001	< 0.001
OKULIX RT Parax	< 0.001	< 0.001	—	< 0.001
OKULIX RT Pup 2.5	0.856	< 0.001	< 0.001	—

Formula	BUII IOLup1D	Olsen ray‐tracing IOLup1D	OKULIX RT Parax IOLup1D	OKULIX RT Pup 2.5 IOLup1D
BUII IOLup1D	—	0.892	0.002	0.003
Olsen ray‐tracing IOLup1D	0.892	—	0.002	0.030
OKULIX RT Parax IOLup1D	0.002	0.002	—	0.002
OKULIX RT Pup 2.5 IOLup1D	0.003	0.03	0.002	—

*Note: p*‐values were obtained from pairwise two‐sample robust *t*‐tests comparing signed prediction errors (SEQ‐PE). Values < 0.05 were considered statistically significant. *p*‐values were adjusted for multiple comparisons (Holm). D, diopters; IOL, intraocular lens; RT, ray‐tracing.

One‐sample robust *t*‐tests revealed that only the Olsen IOLup1D formula showed no significant deviation of the arithmetic prediction error from zero (*p* = 0.65), indicating the highest trueness. In contrast, BUII IOLup1D as well as both OKULIX IOLup1D approaches demonstrated significant systematic prediction errors (all *p* < 0.001).

Precision differed significantly between the formulas. Pairwise comparisons demonstrated significant differences in the variability of prediction errors between most methods. BUII IOLup1D and OKULIX 2.5‐mm pupil IOLup1D were comparable in regard to precision (*p* = 0.20), whereas both were significantly more precise than Olsen IOLup1D and OKULIX paraxial IOLup1D (all adjusted *p* ≤ 0.003). Olsen IOLup1D exhibited the lowest precision, with significantly greater variability compared with all other formulas.

## 4 Discussion

The postoperative hyperopic shift observed after triple DMEK procedures, as confirmed in the present study, is a well‐known challenge in IOL power calculation [2, 5, 6, 20, 21, 22, 23]. In the present study, biometric measurements were primarily obtained by Pentacam AXL devices, with supplemental axial length measurements from IOLMaster 700 when required. Although ray‐tracing‐based formulas theoretically account for individualised corneal geometry and posterior corneal changes, they did not outperform the widely used modern vergence‐based formula in this large cohort. Despite altered corneal anatomy in FECD, conventional formulas demonstrated equal or superior accuracy, questioning the added clinical value of ray‐tracing approaches in routine refractive planning for combined cataract surgery and DMEK.

Alnawaiseh and associates [10] published that in combined cataract and DMEK surgery, the altered anterior–posterior corneal curvature relationship significantly affects refractive prediction and conventional formulas often differ from ray‐tracing predictions, underscoring the need for method‐specific evaluation. It is of note that a change of the posterior curvature would need to be drastic to explain a hyperopic shift of 0.75 D; it is therefore unlikely that the reason is found in the posterior curvature alone. A factor potentially explaining the lower comparatively hyperopic PE observed in the present study compared with previous works may be related to Scheimpflug‐based technology (Pentacam AXL) as the primary source of keratometric detection (Table 3). Several earlier studies investigating IOL power calculation in triple DMEK eyes relied primarily on optical biometres such as the IOLMaster, which use different measurement principles and corneal power models. In a post hoc comparison, we found no significant difference in CCT between Pentacam and IOLMaster 700 measurements, whereas mean keratometry was significantly steeper with the IOLMaster (mean difference 0.20 D). Steeper keratometry values are expected to result in lower calculated IOL powers and consequently a higher risk of postoperative hyperopia. Therefore, differences in biometric technology and corneal power should be considered when comparing refractive outcomes across studies; they may contribute to the relatively smaller hyperopic offset observed in the present cohort.

Knutsson and associates [24] showed that optimisation of IOL constants reduces MedAEs and MAEs and decreases postoperative hyperopic shift by minimising systematic bias. The improvement reported after constant optimisation is comparable to the accuracy observed in our study after adjustment using the IOLup1D method, particularly for BUII and Olsen. However, the optimised constants were derived from a small sample size of only 37 eyes. Given the limited number of triple DMEK cases, robust constant optimisation remains challenging, and the generalisability of such optimised constants to broader populations is therefore limited.

The present study demonstrates that most evaluated formulas, for example, BUII, Olsen ray‐tracing and OKULIX ray‐tracing, displayed a high degree of trueness after a combined triple DMEK surgery. Interestingly, only the OKULIX paraxial ray‐tracing model achieved statistical neutrality with respect to systematic bias. However, this did not translate into superior overall refractive accuracy, suggesting that elimination of systematic error alone is insufficient to improve clinical predictability in this complex surgical setting. In contrast, the pupil‐based OKULIX method exhibited a significant deviation from zero, reflecting a systematic refractive shift. BUII demonstrated significantly lower absolute prediction error than Olsen ray‐tracing.

Across all formulas, median absolute prediction errors ranged from approximately 0.6 to 0.9 D, consistent with the challenging refractive predictability in eyes undergoing combined cataract surgery and DMEK. With respect to clinically relevant refractive thresholds, more than two‐thirds of eyes were within ±1.00 D of the targeted refraction for BUII and OKULIX 2.5‐mm pupil, while the proportion was lower for both the Olsen and OKULIX paraxial method. At broader thresholds, BUII consistently achieved the highest proportion of eyes within ±1.50 D and ±2.00 D.

These findings are in line with previous reports demonstrating that modern commonly used formulas, such as BUII, maintain robust refractive performance, especially in complex corneal conditions, whereas ray‐tracing approaches do not necessarily confer superior accuracy. Our results extend these observations to a triple DMEK cohort, highlighting that stable, well‐validated formulas may outperform theoretically more useful methods in this setting.

A major strength of this study is the use of a single type of IOL model, which reduces confounding effects related to lens design. In addition, all procedures were performed by two experienced surgeons using standardised surgical techniques, thereby minimising inter‐surgeon variability. This methodological consistency enhances the reliability of the results and facilitates their translation into clinical practice.

However, our study has several limitations. First of all, the retrospective design may be associated with inherent bias. Eyes with incomplete preoperative or postoperative data were excluded to ensure that all required parameters were available for each IOL calculation formula, which may have introduced selection bias. Moreover, the absence of subgroup analysed by axial length, including myopic and hyperopic eyes, limits further interpretation, as axial length has been shown to affect refractive prediction accuracy. Differences in axial length measurement algorithms (swept‐source OCT vs. partial coherence interferometry) may result in small systematic offsets, particularly in edematous or irregular corneas. Although such effects are expected to be minimal, they cannot be completely excluded as a source of measurement noise in this retrospective analysis. In addition, several methodological considerations should be taken into account when interpreting the results. The study did not account for postoperative changes in posterior corneal curvature or corneal remodelling over time, which may further influence refractive outcomes after DMEK. No formula‐specific IOL constant optimisation was performed in the present study. Although constant optimisation may reduce systematic bias and improve absolute prediction error, its validity in the setting of triple DMEK remains uncertain. Postoperative refractive outcomes in these eyes are influenced not only by biometric variability but also by corneal remodelling and changes in posterior corneal curvature, which may not be fully captured by simple constant adjustments. Furthermore, triple DMEK represents a relatively uncommon and heterogeneous surgical scenario, limiting the robustness and generalisability of optimised constants derived from single‐centre cohorts. Constants optimised in small datasets may artificially improve performance within the study population but may not be transferable to broader clinical settings. Therefore, the present analysis intentionally reflects real‐world, non‐optimised clinical performance. In this context, the pragmatic IOLup1D adjustment strategy may represent a more clinically applicable approach than formula‐specific constant optimisation. The analysis was constrained to established ray‐tracing formulas and the BUII, as the latter had the lowest MedAE of the 11 formulas examined in a recent published study by Sapok et al. [5]. Consequently, more modern formulas were not considered in the present study. Finally, the lack of subgroup analyses according to axial length or corneal morphology precludes conclusions about formula performance in specific biometric subgroups.

In conclusion, for eyes undergoing the triple DMEK procedure face ongoing challenges in achieving accurate IOL power calculations due to postoperative corneal changes and the associated hyperopic shift. Among the formulas evaluated in our study, the BUII formula demonstrated the most reliable refractive performance, offering high accuracy and low variability, while ray‐tracing approaches did not consistently enhance refractive outcomes. Adjustment strategies such as IOLup1D appear to reduce systematic bias and could serve as practical alternatives to IOL constant optimisation in this complex surgical context. These findings support using robust, well‐validated formulas like BUII for refractive planning in patients undergoing combined DMEK and cataract surgery.

## Funding

The authors have nothing to report.

## Conflicts of Interest

K.P.K.: Lecturing for Oculus and he was supported by an ESCRS Peter Barry Fellowship Grant. J.A.W.: Personal fees from Alcon, Zeiss, Bausch and Lomb, Heidelberg Engineering. T.K.: Consultant, Research and Lecturing for Alcon, Oculus, Schwind, Staar. Consultant and Lecturing for Tarsus, Ziemer. Research and Lecturing for Teleon Surgical. Consulting for Abbvie, Geuder, LensGen, Santen, Stadapharm, Thieme, Zeiss Meditec. Lecturing for Allergan, Bausch & Lomb, Johnson & Johnson, MedUpdate, streamedup. J.O.W., H.B.D., and I.S. have no financial and non‐financial conflicts of interest to declare.

## Supporting information


**Data S1:** Supporting Information.

## Data Availability

The data that support the findings of this study are available on request from the corresponding author. The data are not publicly available due to privacy or ethical restrictions.
