# Supplementary material for: Ray‐Tracing‐Based Intraocular Lens Power Calculation in Combined Cataract Surgery and Descemet Membrane Endothelial Keratoplasty
Source: Clin Exp Ophthalmol. 2026 Jun 23;54(6):769–78. doi: 10.1111/ceo.70135 (PMC13462375; doi:10.1111/ceo.70135)
Supplement: Supplementary file 1 [file CEO-54-769-s001.docx]

**SUPPLEMENTARY MATERIAL**
